# ScipionTomo: Towards cryo-electron tomography software integration, reproducibility, and validation

**DOI:** 10.1016/j.jsb.2022.107872

**Published:** 2022-06-02

**Authors:** J. Jimenez de la Morena, P. Conesa, Y.C. Fonseca, F.P. de Isidro-Gómez, D. Herreros, E. Fernández-Giménez, D. Strelaka, E. Moebel, T.O. Buchholz, F. Jug, A. Martinez-Sanchez, M. Harastani, S. Jonic, J.J. Conesa, A. Cuervo, P. Losana, I. Sánchez, M. Iceta, L. del Cano, M. Gragera, R. Melero, G. Sharov, D. Castaño-Díez, A. Koster, J.G. Piccirillo, J.L. Vilas, J. Otón, R. Marabini, C.O.S. Sorzano, J.M. Carazo

**Affiliations:** a National Center of Biotechnology (CNB-CSIC), Madrid, Spain; b Masaryk University, Brno, Czech Republic; c Inria Rennes - Bretagne Atlantique, Rennes, France; d Max Planck Institute of Molecular Cell Biology and Genetics (MPI-CBG), Germany; e Center for Systems Biology Dresden (CSBD), Germany; f Fondazione Human Technopole, Milan, Italy; g University of Oviedo, Department of Computer Sciences, Oviedo, Spain; h Health Research Institute of Asturias (ISPA), Oviedo, Spain; i Structural Studies Division, MRC Laboratory of Molecular Biology, Cambridge, United Kingdom; J BioEM Lab, Biozentrum, University of Basel, Basel, Switzerland; k University of Leiden, Ultrastructural and Molecular Imaging, Leiden, the Netherlands; l Alba Synchrotron - CELLS (ICTS), Barcelona, Spain; m Superior Polytechnic School. Univ. Autónoma of Madrid. Madrid, Spain; n IMPMC-UMR 7590 CNRS, Sorbonne Université, MNHN, Paris, France

## Abstract

Image processing in cryogenic electron tomography (cryoET) is currently at a similar state as Single Particle Analysis (SPA) in cryogenic electron microscopy (cryoEM) was a few years ago. Its data processing workflows are far from being well defined and the user experience is still not smooth. Moreover, file formats of different software packages and their associated metadata are not standardized, mainly since different packages are developed by different groups, focusing on different steps of the data processing pipeline.

The Scipion framework, originally developed for SPA (de la Rosa-Trevín et al., 2016), has a generic python workflow engine that gives it the versatility to be extended to other fields, as demonstrated for model building (Martínez et al., 2020). In this article, we provide an extension of Scipion based on a set of tomography plugins (referred to as ScipionTomo hereafter), with a similar purpose: to allow users to be focused on the data processing and analysis instead of having to deal with multiple software installation issues and the inconvenience of switching from one to another, converting metadata files, managing possible incompatibilities, scripting (writing a simple program in a language that the computer must convert to machine language each time the program is run), etcetera. Additionally, having all the software available in an integrated platform allows comparing the results of different algorithms trying to solve the same problem. In this way, the commonalities and differences between estimated parameters shed light on which results can be more trusted than others. ScipionTomo is developed by a collaborative multidisciplinary team composed of Scipion team engineers, structural biologists, and in some cases, the developers whose software packages have been integrated. It is open to anyone in the field willing to contribute to this project.

The result is a framework extension that combines the acquired knowledge of Scipion developers in close collaboration with third-party developers, and the on-demand design of functionalities requested by beta testers applying this solution to actual biological problems.

## 1 Introduction

During the last decade, structural biology has witnessed an authentic revolution that has pushed cryoEM and cryoET towards new paradigms with the elucidation of structures at higher and higher resolutions (Smith and Rubinstein, 2014). The reasons behind this growth are the recent advances in sample preparation, instrumentation (better microscopes and the introduction of direct electron detectors), hardware (more computational capabilities), and software.

Until now, SPA has benefited from these advances given the increment in the number of deposited maps in EMDB (Lawson et al., 2016) with resolutions of 3 Å or better. CryoET has also taken advantage of this revolution, but it has been a step behind in this regard. This is caused by inherent problems of the cryoET acquisition technique that requires dealing with low Signal-to-Noise-Ratio (SNR) images affected by the missing wedge problem and with a difficult correction of the CTF (Wan et al., 2016; Fernandez, 2012) complicating the image processing. Moreover, the 3D nature of cryoET data, makes its workflow to be highly computationally demanding and requires interoperability and analysis of the intermediate results at the different steps. Fortunately, this situation is changing, and many developers, who traditionally worked on SPA methods, are now focussing their efforts on the development of new and better image processing methods for cryoET.

There is a broad variety of software for cryoET: Imod (Mastronarde and Held, 2017), Dynamo (Castaño-Díez et al., 2012), Eman (Chen et al., 2019), Relion (Bharat and Scheres, 2016), Xmipp (de la Rosa-Trevín et al., 2013), PySeg (Martinez-Sanchez et al., 2020), emClarity (Himes and Zhang, 2018), Peet (Heumann et al., 2011), PyTom (Hrabe et al., 2012) and M (Tegunov et al., 2021) to list a few. Only some of them can cover the whole processing pipeline. Some of them provide some export/import functionality, like M that can generate star files ready to be used with Relion, or in some cases, there are third-party scripts to do the same for other packages. These kinds of integrations are important but limited in scale, usually interfacing only one or 2 software packages and in some cases through scripting, losing all the traceability. To use the rest, files need to be moved around the filesystem, in some cases creating specific metadata files manually or programmatically, and make extensive use of the command line. Additionally, one will need to deal with the installation of each of the software packages. Even if one manages to handle all this heterogeneity, for the sake of traceability and bookkeeping, all the steps done will require a strict annotation mechanism to be able to report them in a future scientific article. Finally, using a single algorithm to estimate any parameter (the angular orientation of a subtomogram, the defocus at a particular region of a tilted image, etc.) does not allow determining if the estimated parameter is approximately correct or not. We may assess its correctness by comparing this estimation to other estimations performed by alternative algorithms solving the same problem. These complications are the ones addressed by ScipionTomo. It provides a common Graphical User Interface (GUI) for all integrated tomography packages. As it has already happened with SPA, traceability, repeatability, workflow design, agility, and all available Scipion functionalities are provided out of the box for cryoET image processing. The main GUIs consist of a graph that connects the different steps of the pipeline, forms to be filled with the parameters of each method, generic and specific result viewers, and finally the capture of all the logging messages written out during execution.

**Fig. 1:**
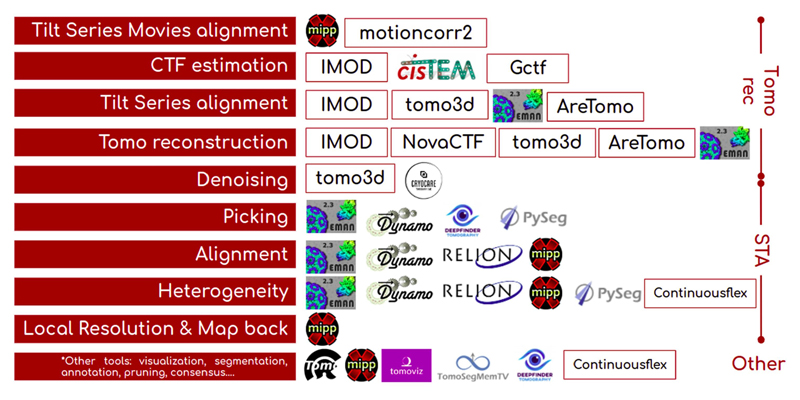
Tomography software currently integrated into Scipion, organized by cryoET data processing pipeline stage (tomography reconstruction, subtomogram averaging, or other tools), and by processing step (from movie alignment to map back).

ScipionTomo covers the complete cryoET pipeline, starting from steps (referred to as protocols hereafter) to directly import the tilt series movies via the mdoc files produced by SerialEM (Mastronarde, 2005), passing through subtomogram averaging (STA), to tilt series refinement protocols. Each of the protocols provides its own help text for each of its parameters and optionally a link to a specific url describing its usage and the related citations for a better understanding. There are currently more than 100 protocols specifically integrated for cryoET from 15 plugins, and more integrations are coming (Fig. 1). In this work, the current state and some highlights of ScipionTomo are described, including:

- Interoperability among all integrated software (with some limitations explicitly mentioned in “Current status and future work” section)
- The ongoing development of protocols for alignment, picking, and STA to get a refined result using the integrated software packages.
- Modern internally developed viewers to cover specific visualization needs in cryoET, like segmentation, oriented 3D coordinates, or tilt series CTF analysis.

From the users’ perspective, they will find a framework in which all the required tools covering the whole data processing pipeline for cry-oET are integrated into the same software environment that has already been used for SPA and model building. Hence, they can forget about:

- Dealing with metadata and image file conversions.
- Managing incompatibilities between different software packages.
- Scripting
- Losing track of the completed processing attempts.
- Using a logbook.

From the software developers’ perspective, Scipion offers a robust and maintained platform where they can integrate their software packages, making them more accessible to the community and offering a better user-friendly experience. A “channel” to reach cryoET users and a versatile installation engine to free them from following, sometimes not easy, installation instructions.

From this point on, the Scipion plugins will be referred to as scipion-em-pluginname, for instance, the imod plugin will be denoted by scipion-em-imod.

## 2 The cryoET pipeline

**Fig. 2:**
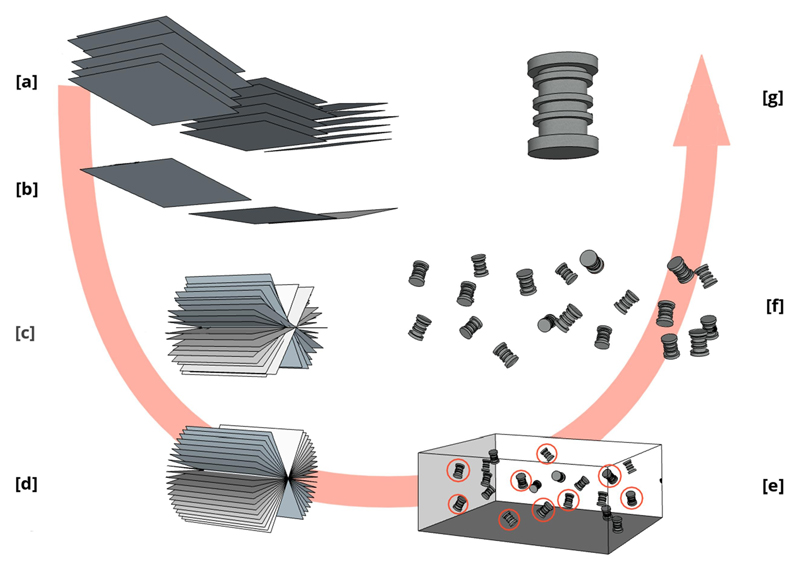
Schematic representation of a “classical” cryoET workflow. Partial tilt series movies (multi-frame images) showing only 3 different tilt angle acquisitions [a]. A beam-induced motion correction method should align all frames to get tilt series images [b]. A hypothetical 19 angles tilt series representation is presented in [c]. Tilt series alignment methods will estimate the transformation matrices to align the whole tilt series and render an aligned tilt series [d]. Tomogram reconstruction methods will generate a tomogram [e]. Tomogram segmentation and picking methods will help to identify 3D coordinates of interest [e - red circles]. Tomogram extraction methods will extract the 3D boxes (subtomograms) containing the particles of interest [f]. STA methods will calculate a final 3D average [g].

In this section, all the steps which compose the cryoET data processing pipeline will be briefly described. With each step, we also briefly present all corresponding protocols in ScipionTomo that can be used. Fig. 2 shows an illustrative overview of the steps which compose a generic cryoET data processing workflow.

### 2.1 Data and metadata import

The first step is to import (registration process) data (images) and/or metadata (information on the images) in Scipion. Both are defined as a ScipionTomo object. These objects are generic representations of each of the data involved in a tomography pipeline, such as “Tilt series”, “Subtomogram”, “CTF”, etc. Scipion offers a set of data import protocols, at least one for each kind of object. Thus, Scipion offers a way to enter at different points of the whole pipeline, starting, for example, from the aligned tilt series if they have been calculated before. Sometimes there is more than one import protocol for the same object, for example for subtomograms. It is possible to load particles obtained with Dynamo (represented with.em files and a Dynamo table), with Relion (expressed as.star files), or any other native file from the integrated packages. Nevertheless, the natural entry point to Scipion is considered to be importing directly the data acquired from the microscope, the so-called “tilt series movies” (a stack of frames acquired for each tilt angle), using the mdoc files generated by SerialEM (The SerialEM Home Page). In the future, other formats like the one produced by Tomo5 TFS (Thermo Fisher Scientific microscopes for electron tomography (Thermo Scientific - Tomography 5 Software) could be supported.

### 2.2 Tilt series movies alignment

Once the tilt series movies have been imported into Scipion, the first step is to align the movies of each angular stack to get the tilt series. This step corrects the beam-induced movement or ice doming due to the interaction of the electron beam with the sample, which results in blurry images when averaging all the frames acquired for the same angle. Two plugins offer a protocol to go through this operation: “tilt series flex align”(Střelák et al. 2020) from scipion-em-xmipptomo and “tilt series motion correction” (Zheng et al., 2017) from scipion-em-motioncorr.

### 2.3 CTF estimation and correction

**Fig. 3:**
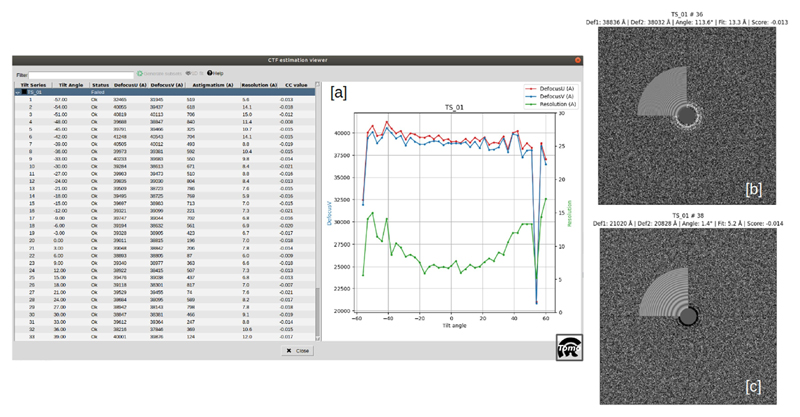
CTF Estimation assistant in Scipion. The main view can be observed in [a], in which the left panel shows the relevant data of each tilt series and its corresponding tilt images, with the corresponding status and the values of the quality indicators calculated, such as the defocus stability through the tilt angle values. The right panel is used to display the corresponding defocus-tilt angle-resolution graph. [b] and [c] correspond, respectively, to an ‘ok’ and ‘no ok’ tilt image result, obtained from images acquired at 48 deg. and 54 deg. (outlier) angles. This CTF view replaces the quality indicators when clicking on a specific tilt image.

This step attempts to model the image formation of the electron microscope, in particular, the defocus and other aberrations that the microscope introduces in the acquired images. The CTF estimation can be carried out with ctffind4 for tilt series (Hu et al., 2019) from the scipion-em-cistem plugin, gctf (Zhang, 2016) from scipion-em-gctf, ctfplotter from the scipion-em-imod plugin, or with scipion-em-emantomo. For CTF objects, the Scipion team has developed a tool called CTF Estimation Assistant (see Fig. 3) which allows the user to determine which of the CTF estimations seems unreliable, by calculating the deviation of the defocusU and the defocusV values for a tilt angle are lower than 20% (default value) respect to the mean value for the tilt series. More complex metrics are planned to be implemented in the future. This tool consists of a dynamic viewer which displays all the data regarding the tilt series on which the CTF has been estimated, with the relevant data and the advice of the assistant per tilt image, as well as the corresponding defocus, tilt angle, and resolution plot. Hence, the manual estimation can be carried out only for the tilt series in which the automatic estimation has not been good enough, improving the efficiency of the data processing. The user can accept or refuse the proposed estimation result, and the ‘good’ and ‘bad’ subsets are directly generated by the CTF assistant.

### 2.4 Tilt series alignment

Then, the tilt series alignment step should be carried out. This step aims to correct the misalignment introduced in the images as a result of tilting the sample (shifts and rotations of the sample holder) from one tilt image to another. The plugin scipion-em-imod offers two approaches for aligning tilt series: automatic tilt series alignment from imod (Mastronarde and Held, 2017) (“tilt series fiducial alignment”, which requires gold bead fiducials in the sample and it is composed of protocols “xcorr pre-alignment”, “generate fiducial model”, and “fiducial alignment”) and manual alignment, which can be done with the “Imod - etomo interactive” protocol where you can use patch alignment and any other functionality eTomo provides. Another protocol for tilt series alignment is the eman “tilt series alignment”, from the plugin scipion-em-emantomo, which, in contrast to imod, does not require fiducials to perform the alignment.

Optionally, the scipion-em-aretomo plugin can also be used to align and reconstruct a tomogram or just provide the alignment parameters for a later reconstruction with other software. Finally, the plugin scipion-em-tomo3d has a protocol that aligns the tilt series and reconstructs the tomogram, called “motion compensated reconstruction” (Fernandez et al., 2019). This protocol also attempts to correct the doming effect in the ice as a consequence of the radiation from one tilt image to the next one. It should be noted that other imod protocols can be used to pre-process a tilt series before the alignment, such as “x-ray eraser”, “dose filter” or “exclude views”, all of them included in the plugin scipion-em-imod.

### 2.5 Tomogram reconstruction

Once the tilt series are properly aligned, they can be reconstructed to obtain the 3D representation of a sample, i.e., a tomogram. Many tomogram reconstruction protocols are integrated into ScipionTomo from the following plugins:

- scipion-em-emantomo: automatic tomogram reconstruction using direct Fourier inversion using fiducials or high contrast regions.
- scipion-em-imod: uses imod WBP (Weighted Back-Projection) based tomogram reconstruction.
- scipion-em-novactf: uses novaCTF (Turoňová et al., 2017) a WBP based tomogram reconstruction with local CTF correction.
- scipion-em-tomo3d: uses tomo3d to execute SIRT (Simultaneous Iterative Reconstruction Technique) based reconstruction or WBP (Weighted Back Projection) based reconstruction (Agulleiro and Fernandez, 2011; Agulleiro and Fernandez, 2015). As it was mentioned before, it also provides a protocol to directly align and reconstruct a tomogram, which uses TomoAlign algorithms (Fernandez et al., 2019; Fernandez et al., 2018; Fernandez and Li, 2021).
- scipion-em-aretomo: it performs both tilt-series alignment and tomogram reconstruction in one protocol (Zheng et al., 2022). The reconstruction can be carried out with SART (Simultaneous Algebraic Reconstruction Technique) or WBP.

The user can choose the protocol that better works for the data which is being processed or even combine several protocols in the same project. For example, SIRT and SART-based methods offer very good contrast for picking, but the particles can be then extracted from the corresponding tomogram reconstructed with the local CTF correction (novaCTF) correction or any other reconstruction algorithm.

### 2.6 Tomogram denoising

An available optional step could be denoising reconstructed tomograms to enhance their visualization. This can be of special interest to visualize cellular environments or to facilitate the identification of particles for a later picking step. Carrying out this step or not, depends mostly on the data being processed: for example, for very small particles, it is recommended not to denoise the tomograms, because the small particles could be removed in the denoising calculations. On the other hand, these kinds of methods may be very useful for cellular environment analysis. But it depends on each dataset. Scipion integrates three denoising algorithms, Tomoeed (Moreno et al., 2018) and Tomobflow (Fernandez, 2009), from plugin scipion-em-tomo3d, and a deeplearning-based approach named cryoCARE (Buchholz et al., 2019), from a plugin with the same name. Note that the last method requires pairs of tomograms reconstructed from the tilt series generated using only the even and odd frames of the tilt series movies, respectively. Supplementary material 1 explains how to get these pairs of even and odd tomograms with ScipionTomo.

### 2.7 Tomogram particle picking

The picking step is the process of identifying the complexes of interest in a tomogram. Here there are two possible scenarios: the first one consists of identifying ‘isolated’ macromolecules while the second one applies to particles that are tethered to a bigger biological entity, like membrane proteins. For the first scenario, ScipionTomo offers four picking protocols, three of which are from the scipion-em-emantomo plugin: manual picking, template matching-based picking, and semiautomated deep-learning-based picking. The fourth protocol is Deep-Finder(Moebel et al. 2021), a semi-automated solution that employs a deep neural network to learn a model for macromolecule species and their cellular environment from annotations. For the second scenario, there are two protocols integrated into ScipionTomo. The “manual vectorial picking” protocol from Dynamo and the picking from PySeg, which is automatic and is based on computing spatially embedded graphs with a previous segmentation and annotation of the membranes. For these operations, ScipionTomo also provides the TomoSegMemTV (Martinez-Sanchez et al., 2014) and membrane annotation protocols respectively, both from the plugin scipion-em-tomosegmemtv and a segmentation protocol from the DeepFinder plugin.

### 2.8 Picking pruning tools

Picking output can be further refined with some post-picking operations, such as “picking consensus”, “remove duplicates”, and “filter by normal” (only for oriented coordinates), which are the protocols offered by the scipion-em-tomoviz plugin. The picking consensus protocol can be used to determine the agreement between different particle picking algorithms. It takes different sets of 3D coordinates obtained from different softwares. These outputs can then be combined to remove false-positively picked particles, using multiple criteria, such as tolerance radius. The “remove duplicates” protocol works on the coordinates that are closer than a given distance threshold. The resulting coordinate is the average of the coordinates which are closer to each other than the provided threshold. In the case of oriented particles, like membrane particles, the directional picking (coordinates and angles) can also be refined using the protocol “filter by normal”. This protocol takes the membrane surface and the particles and filters them by different criteria related to the direction normal to the membrane, such as a specified tilt angle or the normal direction of the coordinate to the vesicle surface, within a specified angular tolerance. If the user has a set of coordinates with orientation, but not the surfaces or meshes corresponding to their membranes, these surfaces can be created from the oriented coordinates by using the protocol “fit vesicles” from the plugin scipion-em-xmipptomo.

Another way of pruning directionally picked particles is by applying 2D structural classification with PySeg. Firstly, 3D subvolumes are flattened by radial averaging along their in-plane (locally tangent to the membrane surface) axis, thus obtaining 2D particle representations with higher SNR and neglecting misalignment around in-plane angles. Secondly, these 2D images are classified into structurally homogeneous groups by the Affinity Propagation algorithm, this algorithm is unsupervised as it does not require the number of input classes, nevertheless, K-means and Hierarchical Clustering can also be used. Finally, the user manually selects the most promising classes based on their 2D averaged appearance (more information in Supplementary material 2).

### 2.9 Subtomogram averaging (STA) and per-particle per-tilt refinement

This section lists the available software methods available to get a final 3D average. There are 2 main approaches: STA and per-particle per tilt refinement. STA deals with subtomogram 3D volumes as input particles whereas a per-particle per-tilt approach goes back to the tilt series to make a finer refinement as described in (Pyle and Zanetti, 2021).

There are three plugins in ScipionTomo that offer both STA and perparticle per-tilt protocols. These are:

- scipion-em-dynamo: it offers protocols for particle extraction, subboxing (locating the position of subunits of interest), subtomogram principal component analysis (PCA), and multireference alignment (MRA), which aligns each particle against a group of different model templates.
- scipion-em-emantomo: it contains another particle extraction protocol, an initial model generation protocol, a subtomogram average refinement protocol, and a per-particle per tilt refinement.
- scipion-em-reliontomo: Relion 4 (RELION) is the new version of the popular and powerful software package for cryoEM, which has expanded its functionality to cryoET. The plugin includes most of the functionality but leveraging the advantages of Scipion to offer a generalized entry point to it, using the data model and conversion functionalities from Scipion to allow the user to go into the scipion-em-reliontomo plugin no matter the software package used to estimate the CTF or to align the tilt series and generating automatically all the required star and any other auxiliary files, such as the order list files. Another advantage of using Relion inside Scipion is the graphical traceability. The workflow proposed by Relion is large and iterative. The solution proposed by Relion consists of using a star file called optimisation_set which tracks the required files from one step to another, but as the number of steps increases, it may be over-whelming to the user. In turn, graphic traceability can provide a clearer perspective and comprehension of the whole procedure. The last advantage is related to another canonical Scipion feature, which is providing a set of visualization tools, hence, the user can use any of them without having to deal with data conversion, compatibility issues, etc.

### 2.10 Heterogeneity

#### 2.10.1 Continuous conformational variability analysis

In cryoET, continuous conformational transitions of biomolecular complexes can be both a huge obstacle to high-resolution subtomogram averaging and a unique opportunity to describe, at once, multiple bio-molecular conformations relevant to the given environment (purified samples or cells in health and disease). HEMNMA-3D (Harastani et al., 2021) and TomoFlow (Harastani et al., 2022) from the scipion-em-continuousflex plugin allow analyzing subtomograms in terms of continuous conformational variability. Both methods represent a given set of subtomograms in a common low-dimensional space based on the motion field obtained between each subtomogram and a given reference. HEMNMA-3D determines the motion field by displacing the reference using normal modes, with the normal-mode amplitudes calculated by matching the subtomogram with the displaced reference. In TomoFlow, the motion field is described by the optical flow between the subtomogram and the reference. For the reference, both methods can use an EM map such as subtomogram average (for normal mode analysis, HEMNMA-3D will internally use a 3D Gaussian-based representation of this EM map) and HEMNMA-3D can optionally use an atomic reference. The obtained low-dimensional conformational space can then be explored in terms of subtomogram averages obtained by interactive grouping of subtomograms with similar conformations (closest points in the densest regions in this space) or in terms of animated displacement of the reference model along the densest regions in this space.

Furthermore, the scipion-em-continuousflex plugin contains methods originally developed for analyzing continuous conformational variability in single-particle cryoEM images (analysis of images with normal modes of a reference by HEMNMA (Harastani et al., 2020; Jin et al., 2014) and for deriving atomic models from cryoEM maps (flexible fitting of an atomic structure into an EM map using NMMD (Vuillemot et al., 2022) that combines normal modes and molecular dynamics simulation). These two single-particle cryoEM methods (HEMNMA and NMMD) could also be used for analyzing 2D tomography data (e.g., projections of subtomograms) and for deriving atomic models from high-resolution subtomogram averages, respectively.

Finally, the scipion-em-continuousflex plugin provides a method for synthesizing subtomograms with or without conformational heterogeneity, which can be used to synthesize data for testing tomography methods under development.

### 2.11 Post-processing

#### 2.11.1 Local resolution estimation

The plugin scipion-em-xmipptomo contains a protocol for estimating the local resolution in tomography, called MonoTomo (Vilas et al., 2020). This protocol extends the SPA algorithm of MonoRes (Vilas et al., 2018) adapted to address the problems specific to tomography, such as the spatially variant noise or the large size of the tomograms. It requires two tomograms, one obtained from a tomogram reconstructed using the even frames of the movies that compose the tilt series and the other one obtained using its corresponding odd frames, called odd and even tomograms. Alternatively, the odd and even tomograms can be reconstructed with the odd and even tilt series images, but this practice is less optimal due to the angular sampling being twice the original one. The odd and even tomograms can be obtained with ScipionTomo as explained in supplementary material 1. Output can be seen in Fig. 5.

An immediate application of local resolution estimations is the possibility of filtering the picked subtomograms according to their local resolution average, as a pruning step. The result of the filter is a set of subtomograms with a given range of local resolution, allowing the selection of the subtomograms with the highest quality. This kind of pruning is implemented in Scipion in the scipion-em-xmipptomo by the “filter coordinates by map” protocol.

#### 2.11.2 Subtomogram subtraction

Subtomogram subtraction is implemented in the plugin scipion-em-xmipptomo as a post-processing tool. This protocol subtracts a subtomogram average from a set of subtomograms, which are internally aligned and numerically adjusted to obtain reliable results (Fernández-Gimenéz et al., 2021). A mask can be provided if the user wants to perform the subtraction within a specific region, for example, to subtract the membrane of a set of particles that are attached to it.

#### 2.11.3 Map back

Once STA is complete, you may want to go back to the tomogram and place the obtained average into the cellular context (Fig. 6). This “map-back” operation can be done with the “map back” protocol from scipion-em-xmipptomo.

### 2.12 Visualization tools

**Fig. 4:**
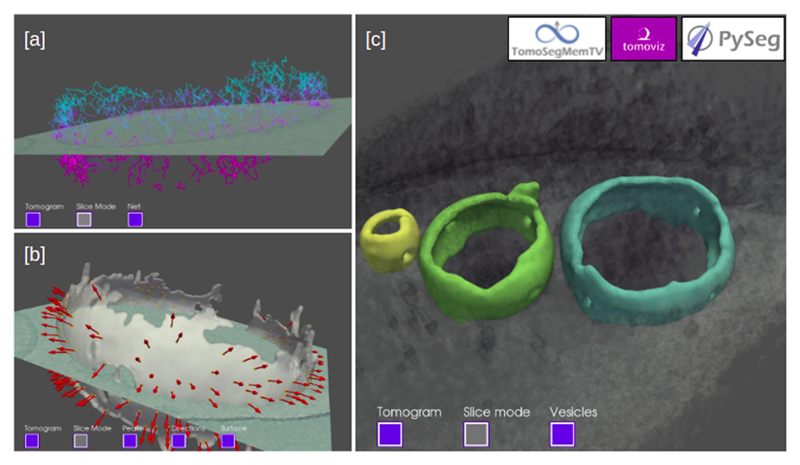
Examples of scipion-em-tomoviz plugin for 3D visualization. PySeg filaments of a vesicle are shown overlapping with a slice of a cropped tomogram [a]. A membrane vesicle showing the orientations of the picked particles overlapping with a slice of a cropped tomogram [b]. The surface of 3 segmented vesicles using TomoSegMemTV rendered on a tomogram [c].

**Fig. 5:**
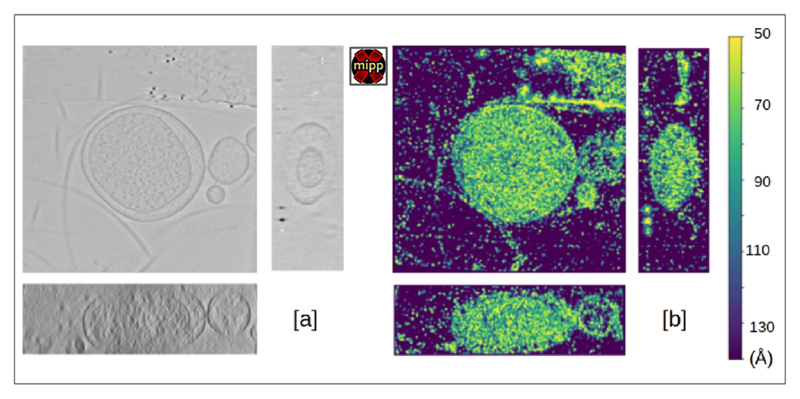
Example of xmipptomo-monotomo estimation of the local resolution of a tomogram reconstructed from TS-G8-box7-001 tilt serie, from the dataset EMPIAR-10364. Showing [a] the Z (top), X (right) and Y (bottom) slice axis and [b] the color scale ranging from 50 to 140 Å resolution.

**Fig. 6:**
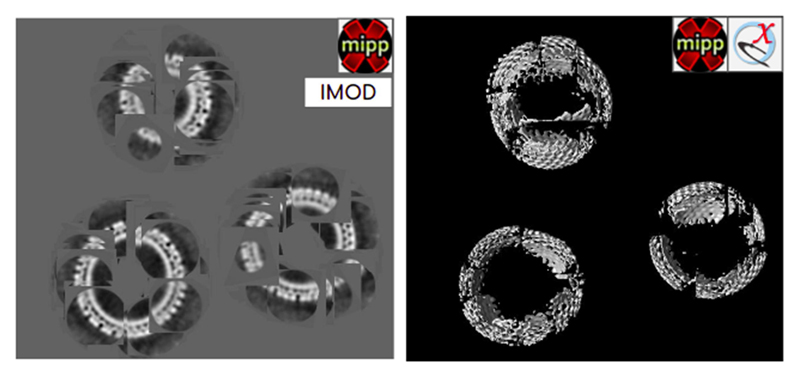
HIV capsids subtomogram average from EMPIAR-10643 mapped back to their native locations in the tomogram using either Imod viewer (on the left) or represented in a 3D space using ChimeraX (on the right).

ScipionTomo offers a complete set of visualization tools, most of them coming from the integrated software, such as IMOD – 3dmod for tilt series and tomograms, DeepFinder and Eman viewers for tomograms and 3D coordinates, xmipp data viewer for tilt series, tomograms and subtomograms to name a few. But there is also a set of internally developed 3D viewers, part of the scipion-em-tomoviz plugin, which can be used to render in a 3D space tomograms, subtomograms, coordinates, normals, vesicles, graphs and filaments. Fig. 4 illustrates some examples of the plugin scipion-em-tomoviz visualization tools.

## 3 Workflow examples

### 3.1 Workflow 1: From tilt series to particle picking

**Fig. 7:**
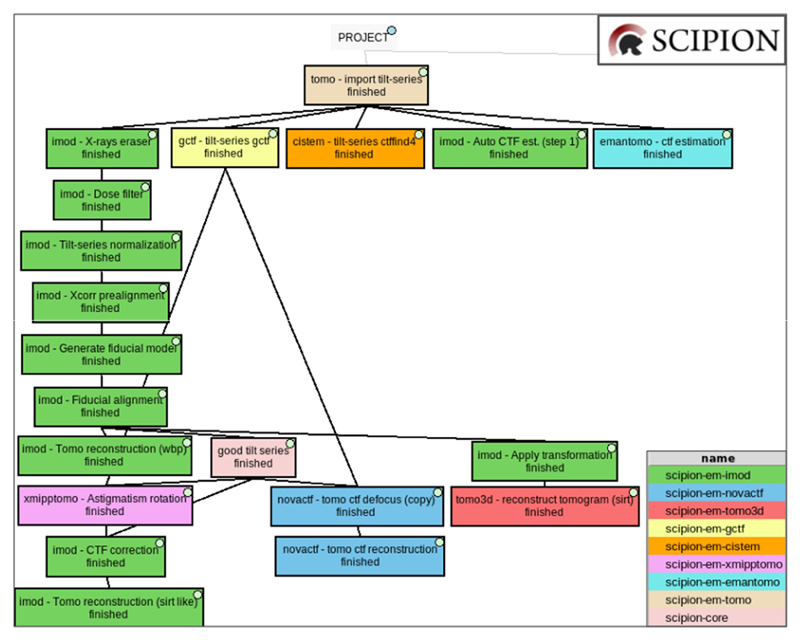
Example of workflow to go from the tilt series to tomograms reconstruction. A different color was used to represent the different plugins used. Different protocols to estimate the CTF (imod, ctffind4, gctf, and emantomo) and reconstruct the tomograms (imod, novaCTF and tomo3d) are shown, together with other preprocessing, auxiliary, or data managing methods, illustrating the interoperability and versatility offered by Scipion.

Fig. 7 illustrates the versatility offered by Scipion and different walkthroughs that can be followed to go, in this case, from the tilt series to the tomogram reconstruction, including the CTF estimation and correction. The steps of the workflow are labeled with a different color for each different plugin is used. Thus, each box represents a different protocol that belongs to a specific plugin. Although the import and the alignment of the raw movie files obtained from the microscope are not included in this workflow, they are fully covered in Scipion, as explained in previous sections.

The current reconstruction workflow was carried out on EMPIAR-10453 (Iudin et al., 2016) dataset, which provides the tilt series and the acquisition information. They were imported into Scipion using the plugin scipion-em-tomo. Focusing now on the fiducial alignment stage, it was mainly carried out using protocols from the plugin scipion-em-imod.

Before aligning the tilt series, they were pre-processed through steps of X-ray erasing, dose filtering, and tilt series normalization (binning). Then, the automatic tilt series alignment was carried out with “xcorr pre-alignment”, the “fiducial model generation”, and the “fiducial alignment” protocols. We recommend attempting the automatic alignment at first, as shown in this workflow because bad alignments can be corrected manually later with the eTomo protocol or equivalent and joined back to the initially well-aligned set of tilt series. Moving back to the fiducial alignment, in this example, we have used two different algorithms to reconstruct tomograms without any CTF estimation from scipion-em-imod and scipion-em-tomo3d. It can be observed that before reconstructing with scipion-em-tomo3d, another protocol from the plugin scipion-em-imod called “apply transformation” is used. This is because Scipion stores the cumulated transformations carried out over the data in a 3D transformation matrix (shifts and angles) to save disk space and avoid artifacts that might be generated due to several interpolations over the same images. The interpolation operation of applying the transformation matrix to the tilt series is performed internally in each protocol that requires it. Thus, this protocol offers the user the possibility to check the quality of the alignment by applying the transformation matrices. Note that although not shown in this example, tilt series alignment could have been done with aretomo or eman instead of imod.

Going now to the CTF estimation, there are different protocols available to carry out this operation. Only the “tilt-series gctf” protocol was used in later reconstructions but any other could have been used. The other estimations have been performed and included in the Fig. 7 to illustrate the versatility and interoperability offered by Scipion.

**Fig. 8:**
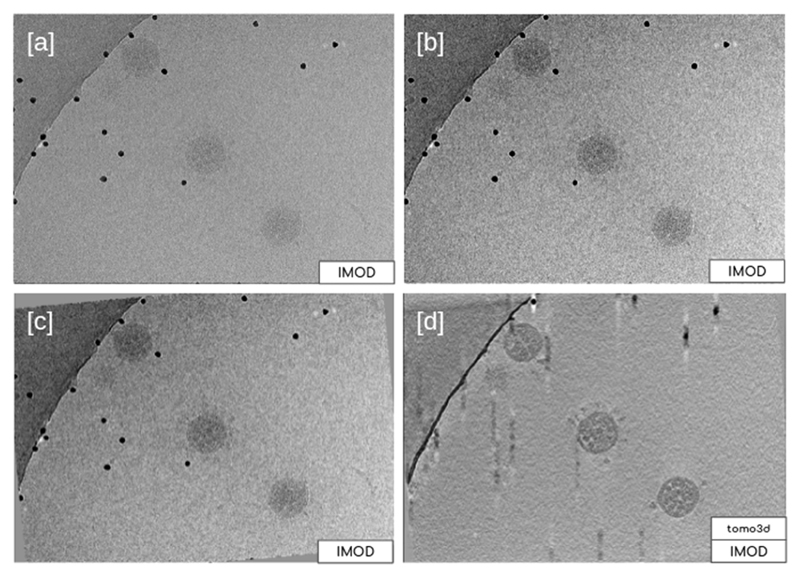
Results obtained for some of the steps carried out to reconstruct TS_293 of dataset EMPIAR-10453 [a] and [b] correspond to the original tilt series and preprocessed tilt series, respectively, being [b] the result of the tilt series X-ray erasing dose filtering, and downsampling to bin 4. Sub-figure [c] is the result of the automatic fiducial alignment offered by plugin scipion-em-imod, and sub-figure [d] is the result of the SIRT reconstruction performed with plugin scipion-em-tomo3d without CTF estimation in this case (it corresponds to the green box named “Imod - Tomo reconstruction (wbp)” in Fig. 7). All the subfigures represent data displayed with viewer IMOD-3dmod.

Now that the CTF has been estimated, it is possible to use this information as an input to other algorithms to obtain a more accurate reconstruction of the final tomogram. There are two main possibilities for this: (i) scipion-em-imod and (ii) scipion-em-novactf. The first one performs a 2D CTF correction of the tilt-series for a posterior reconstruction that can be used with any other classical reconstruction algorithm offered by ScipionTomo. The second and more accurate option performs a 3D CTF corrected reconstruction, which corrects the CTF during the reconstruction process, considering the coordinates of each voxel, rendering results like those shown in Fig. 8.

### 3.2 Workflow 2: Directional picking

**Fig. 9:**
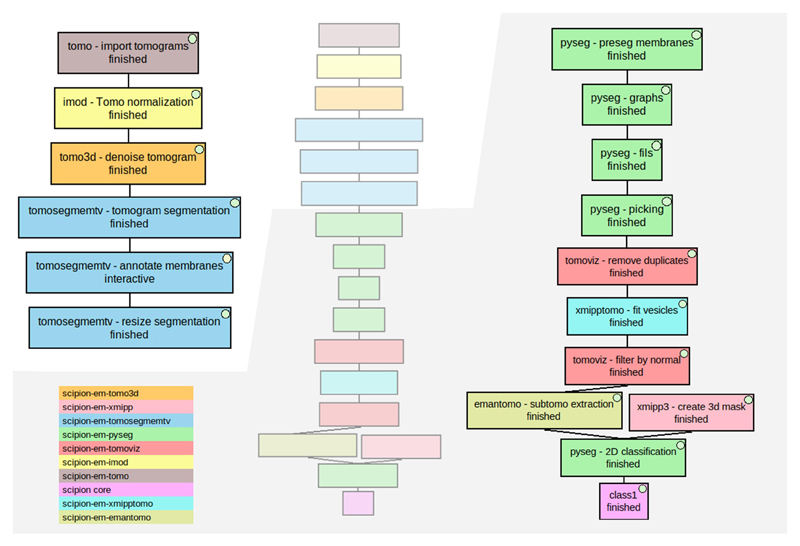
Directional picking workflow to pick membrane-associated ribosomes of an in situ tomogram (EMD-10439). The workflow in the middle represents the whole workflow, while the images on both sides are zoomed-in parts of it to provide better visualization. Different colors were used to represent each plugin involved. The color legend is on the bottom left. The different stages described in the text are (i) boxes named “tomo - import tomograms” (in brown), “Imod - Tomo normalization” (in yellow), and “tomo3d - denoise tomogram” (in orange), (ii) the 3 blue boxes named “tomosegmemtv - tomogram segmentation”, “tomosegmemtv - annotate membranes” and “tomosegmemtv - resize segmentation”, (iii) the green boxes named “pyseg - preseg membranes”, “pyseg - graphs”, “pyseg - fils” and “pyseg - picking”, (iv) boxes named “tomoviz - remove duplicates” (in red), “xmipptomo - fit vesicles” (in light blue) and “tomoviz - filter by normal” (in red), and (v) boxes named “emantomo - subtomo extraction” (in dark yellow), “xmipp3 - create 3d mask” (in light red), “pyseg — 2D classification” (in green) and “class1” (in pink).

In this section, we show a workflow that illustrates how to pick membrane proteins (in this case membrane-associated ribosomes) using segmentation, annotation, and directional picking protocols inside the Scipion framework (see Fig. 9). It was carried out on the dataset EMD-10439 (Lawson et al., 2016). Different colors have been used for different plugins to illustrate the interoperability, traceability, and customizability offered by Scipion. The small diagram in the center is the whole workflow, which has been split into two halves for visualization purposes.

We can group the protocols that compose this workflow into five stages: (i) data preparation, (ii) membrane segmentation, (iii) directional picking, (iv) particle filtering, and (v) particle extraction.

The first stage, data preparation, involves importing the tomograms with the plugin scipion-em-tomo and binning them with the protocol “imod - Tomo normalization” from the plugin scipion-em-imod. The binning was carried out to reduce the execution time of the posterior denoising, segmentation, and annotation steps. At this stage, the tomogram is also denoised with the plugin scipion-em-tomo3d.

The second stage is carried out with the plugin scipion-em-tomosegmemtv. In the first step, the membranes are segmented and then manually annotated using the Membrane Annotator tool, which is part of the same plugin. Finally, the segmented and annotated data are resized to their previous size for the correct picking of membrane particles.

The third stage is composed of four steps of PySeg directional picking, each performed by a different protocol of the scipion-em-pyseg plugin. The first step is the “preseg”, which is used to annotate each vesicle membrane and the inner and outer surroundings. Then, the graphs are calculated and refined into the filaments, which are finally used for the picking.

The fourth stage corresponds to the picking pruning. 3D coordinates are first grouped into geometrical related ellipsoids with “fit vesicles” leaving out those far from the geometry, from the scipion-em-xmipptomo plugin. Later, the “filter by normal” protocol, from the scipion-em-tomoviz plugin, removes any oriented coordinate whose normal is tilted more than N degrees from the perpendicular to the membrane.

**Fig. 10:**
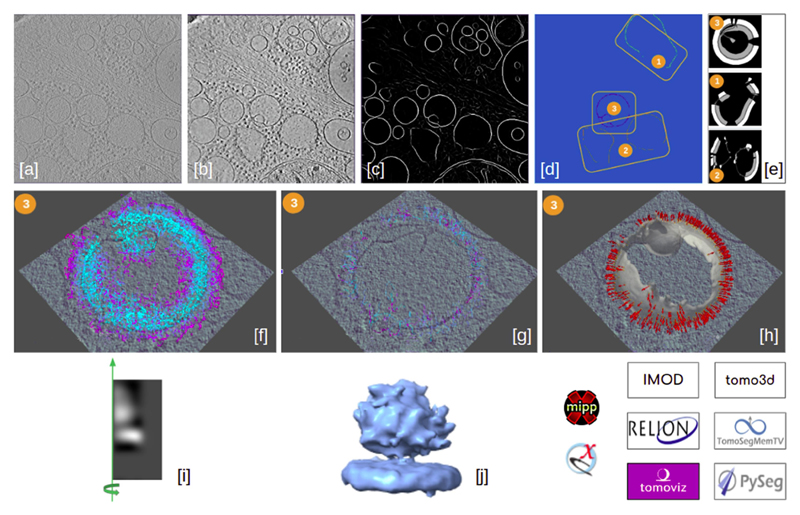
Graphical walkthrough over directional picking results: imported tomogram displayed with IMOD-3dmod [a], denoised tomogram with the plugin scipion-em-tomo3d and displayed with IMOD-3dmod [b], membrane segmentation with the plugin scipion-em-tomosegmemtv, displayed with IMOD-3dmod [c] and manual annotation of the segmented membranes, carried out and displayed with the Membrane Annotator tool from the plugin scipion-em-tomosegmemtv [d]. Sub-figures [e] to [h] show the results of the preseg, graphs, filaments, and picking, respectively, steps of the directional picking procedure with PySeg. Preseg results in [e] are displayed with xmipp-Dataviewer and the rest, for the membrane labeled as 3, with viewer tomoviz from the plugin scipion-em-tomoviz. Sub-figure [i] represents the best 2D class obtained with the 2D classification from the plugin scipion-em-pyseg, represented as a rotational average around the green axis (which is why the class representative is half of the particle box). Sub-figure [j] shows a volume resulting from averaging picked particles after some STA steps (carried out with the plugin scipion-em-reliontomo, Relion 3 version) in ChimeraX viewer (contained in the plugin scipion-em-chimera).

The final stage is the particle extraction, made with the scipion-em-emantomo plugin, and the 2D classification carried out with the scipion-em-pyseg plugin. The classification protocol requires a mask to calculate a rotational average in the desired area where the particle is located. A cylinder mask was created with the scipion-em-xmipp plugin to cover the particle, the membrane, and a small part of the inner surroundings. Finally, a subset of the particles that belongs to the best class was selected using the “Xmipp Dataviewer”. Intermediate results can be seen in Fig. 10.

### 3.3 Workflow 3: Subtomogram averaging

**Fig. 11:**
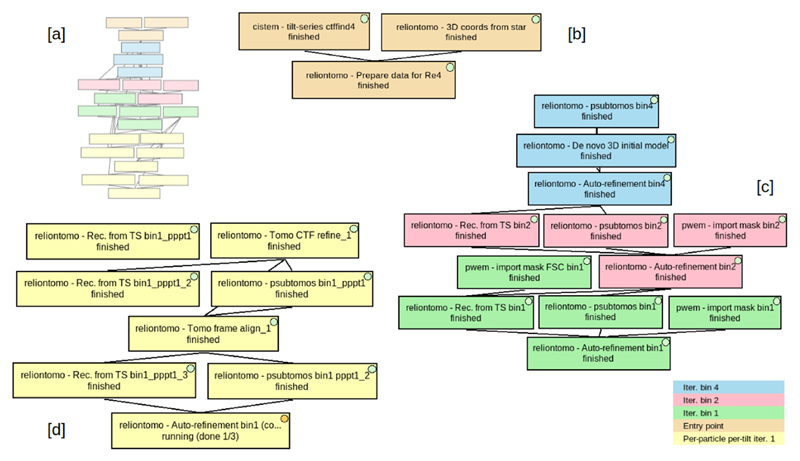
STA workflow to get a model of the HIV capsid following Relion 4 tutorial (EMPIAR-10164). Different colors were used to differentiate the three stages proposed, being [a] the whole workflow structure, [b] the data generation and entry point to Relion 4 for tomography, [c] the three iterations, from binning 4 to binning 2, and finally binning 1 of the stage that takes from the tilt series and coordinates to the subtomogram volume, and [d] the first iteration of the so-called tomo refinement cycle.

The dataset used for this STA workflow corresponds to the one used in the STA tutorial proposed in the documentation of Relion4 (Subtomogram tutorial). It goes from the tilt series and particle coordinates to the final model, computing iterative particle reconstructions and refinements from bin 4 to bin 1. At that point, the so-called “per particle per tilt” (pppt) methodology is applied to improve the resolution of the model. The dataset is a subset of EMPIAR-10164, composed of the five tilt series named TS_01, TS_03, TS_43, TS_45, and TS_54. The tutorial dataset also provides the TS alignment data estimated with IMOD-etomo, the CTF estimation is performed with ctffind4 and the coordinates are picked, but without specifying the picking algorithm used. From Scipion, it is possible to take advantage of part of the precomputed data or to start from the tilt series movies and carry out all the steps required until the tilt series alignment, the CTF estimation, and the particle picking. As can be observed in Fig. 11, the CTF estimation was calculated with the protocol tilt series ctffind4 from the plugin scipion-em-cistem, using the same parameters as those in the defocus files provided with the dataset (this step of CTF recomputing is included just to further show Scipion versatility).

Once all the data is imported and/or generated into Scipion, the first step of the Relion4 workflow is covered by protocol “prepare data for Relion 4”, which will generate the input to relion4 given the CTF estimation, the unaligned tilt series, the alignment transformation data, and the picked coordinates. The tilt series should be at bin 1, and the given coordinates are scaled according to the ratio of the sampling rate of the tomograms in which the coordinates were picked, in case they were at a different binning. To get the final model at bin 1, the tutorial proposes a three iteration loop decreasing the binning factor from 4 to 2 and finally to 1. Each iteration step, as shown in Fig. 11, consists of generating the so-called pseudo-subtomograms, which are subtomograms generated directly from the tilt series. The refined pseudo-subtomograms are then used to reconstruct the particle decreasing the binning and, after that, the next iteration begins repeating those steps with the new binning factor. The tutorial dataset provides some centered masks at different binning factors which can be used as a reference to estimate the Z shift that may be necessary to be applied to the refined pseudosubtomograms. Scipion core offers a protocol named “edit set” that can be used to carry out this operation.

**Fig. 12:**
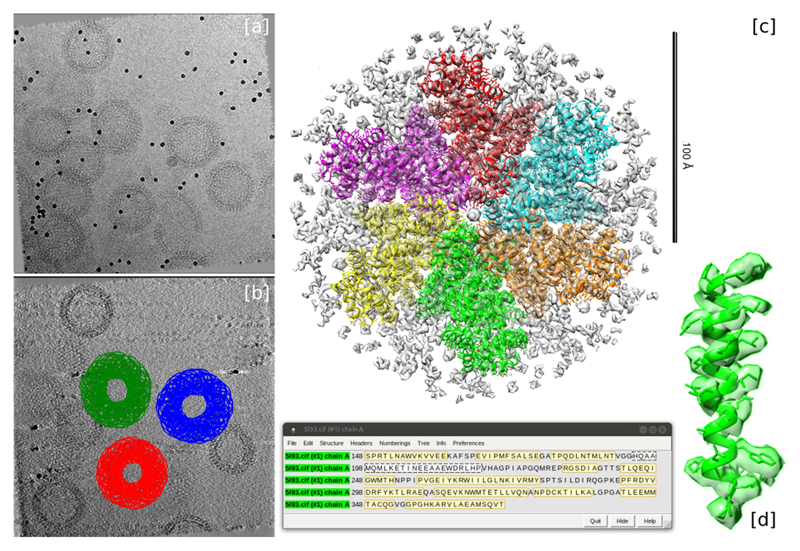
Intermediate results of the STA workflow to get a model of the HIV capsid following Relion 4 tutorial (EMPIAR-10164), being [a] the tilt series named TS_01 displayed with IMOD-3dmod viewer, [b] the picking of some membrane particles from the same tilt series, displayed with EMAN, [c] and [d] the top and the side view of the volume obtained with the auto-refinement from Relion 4 at bin 1, displayed with ChimeraX as surfaces, and [e] and [f] the same views, but corresponding to the auto-refinement with which the first iteration of the tomo refinement cycle finishes, displayed with ChimeraX as a tilted slab.

Once we have obtained a model at bin 1, the so-called tomo refinement cycle can be carried out. It consists of multiple iterations of the pppt protocols, following the sequence: reconstruction at bin 1 with an FSC mask (provided) to generate an FSC reference apart from the reconstruction, CTF refinement, make a new set of pseudosubtomograms considering the refined defocus, reconstruct again to get new halves and an improved FSC reference and frame alignment to generate the particles motion data. The cycle iteration finishes with another generation of pseudo-subtomograms and another reconstruction whose results will be used to feed an auto-refine protocol. The loop can be repeated as many times as desired until the resolution stops increasing. Intermediate results are shown in Fig. 12.

### 3.4 Workflow 4: The “SPA leap”

**Fig. 13:**
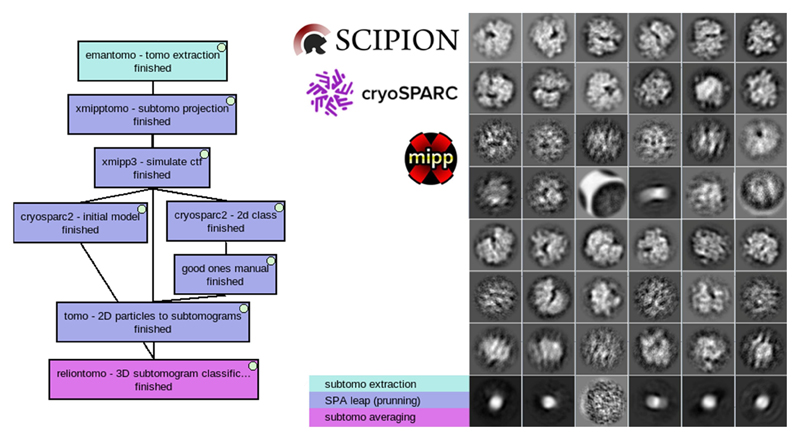
“SPA pruning leap” workflow and results for EMPIAR-10045 dataset. The workflow shows the initial model and 2d classification SPA methods from cryosparc (left). 2d classes obtained during 2d classification, some resembling ribosomes others not (right).

Given the maturity of the existing methods of SPA and the integration of most of them in Scipion, it is very easy to make use of SPA picking and pruning methods. In this example (Fig. 13) we have used EMPIAR-10045: Aligned tilt series images of *S*. *cerevisiae* 80S ribosomes. Any 3D picker will produce false positives, which have to be removed. It is here where we made a “SPA leap” to prune and get an initial volume. “xmipp subtomo projection” from the scipion-em-xmipptomo plugin was used to project all the subtomograms on their Z-axis and generate the same number of 2D particles. Then, we added a zero defocus CTF with “xmipp3 simulate CTF” as CTF parameters are requested by cryosparc “2D classification” (Punjani et al., 2017) and “ab-initio” protocols. Particles from the “good classes” were manually selected with “Xmipp data viewer” producing the “good ones manual” set that was used to prune the original set of subtomograms using the “tomo – 2D particles to subtomograms” protocol. Now, back into the 3D space, a “relion 3D classification” was used to get all the pruned subtomogram set aligned into a single class, feeding it with the initial volume generated by cryosparc in the SPA pruning leap. Additionally, another SPA leap can be made to use SPA picking methods as explained in supplementary material 3.

## 4 A collaborative software project

ScipionTomo is part of the bigger project “Scipion” that aims to provide a free, open-source, high-quality smart workflow engine. Many developers are contributing to this project and everyone is welcome to join. Beta-testers are also an important part, as they are carefully processing actual datasets and providing critical feedback for future stable releases. Scipion can be extended through plugins. At any time, any software developer can create a plugin and add more tools to this catalog, and therefore more possibilities and options to get the best possible result. This is the case for Deepfinder, cryoCARE, pySeg, and Tomo-SegMenTV, where their developers’ close collaboration has been key in providing the best possible integration.

## 5 Current status and future work

To this date, we consider ScipionTomo is in Beta state. This means is stable enough to be carefully relesed upon request. We welcome any betatester volunteer and we offer direct communication to all the developers involved through our slack workspace. Although most of the pipline is covered we are currently focused on per particle per tilt methods (Relion 4, eman). One limitation we currently face is the inability to mix per particle per tilt methods from different software. Providing a reference intial model from eman to relion or viceversa should not be an issue, but mixing steps is still not possible. Additionally we need to tests the pipeline when special cases occur during reconstructions like “views exclusion”, “”offset reconstructions” and later on pppt approaches are used.

Currently development efforts are the following:

- Streaming mode (outputting results on the fly), which would allow the facilities to evaluate the results during the acquisition.
- Aitom (Zeng and Xu, 2019) integration is being co-developed with its developers
- memBrain (Lamm et al. 2022) integration is being co-developed with its developers
- surfaceMorphometics(Barad et al. 2022) integration is being co-developed with its developers
- emClarity and novaSTA (turonova. turonova/novaSTA: novaSTA., 2020) are being integrated.
- Organizing more courses and tutorials, see supplementary material 4 for available training material.

## 6 Conclusions

In a fast-growing domain with quickly evolving techniques, software engineers do not wait for standards. Thus, the software is produced in an isolated manner and cryoET is no exception. In this paper, we presented ScipionTomo, a framework that improves the usability and interoperability of many software packages currently available for cryoET data processing. ScipionTomo is designed to be the glue to bring many of the available software together, allowing the structural biologist to focus on a research problem and avoiding completely the need for manual data conversion, programming, or scripting. As a consequence of the integration, Scipion is naturally annotating all the steps done during the tomography image processing, once more freeing the user from documenting all the steps done during the image processing. As an extensible framework, any future or not yet integrated software can be plugged in and join the ScipionTomo ecosystem. To date, ScipionTomo is open upon request for beta testing and has not been widely released yet. Currently, all steps of the cryoET data processing workflow are covered with several options for each of the steps rendering accurate results.

## Supplementary Material

Supplementary Data
