# Supplementary material for: ScipionTomo: Towards cryo-electron tomography software integration, reproducibility, and validation
Source: J Struct Biol. Author manuscript; Available in PMC 2022 Sep 22. (PMC7613607; doi:10.1016/j.jsb.2022.107872)
Supplement: Supplementary Data [file EMS154348-supplement-Supplementary_Data.docx]

| **#** | **Plugin** | **Protocol** |
| --- | --- | --- |
| 1 | aretomo | aretomo - tilt-series align and reconstruct |
| 2 | cistem | cistem - tilt-series ctffind4 |
| 3 | continuousflex | continuousflex - convert to pseudoatoms |
| 4 | continuousflex | continuousflex - nma analysis |
| 5 | continuousflex | continuousflex - nma alignment vol |
| 6 | continuousflex | continuousflex - nma vol dimred |
| 7 | continuousflex | continuousflex - nma vol cluster |
| 8 | continuousflex | continuousflex - refine subtomogram alignment |
| 9 | continuousflex | continuousflex - tomoflow protocol |
| 10 | continuousflex | continuousflex - tomoflow dimred |
| 11 | continuousflex | continuousflex - tomoflow vol cluster |
| 12 | continuousflex | continuousflex - subtomogram averaging |
| 13 | continuousflex | continuousflex - classify subtomograms |
| 14 | continuousflex | continuousflex - apply subtomogram alignment |
| 15 | continuousflex | continuousflex - synthesize subtomograms |
| 16 | continuousflex | continuousflex - missing wedge restoration |
| 17 | continuousflex | continuousflex - volume denoise |
| 18 | cryocare | cyocare – Load Model |
| 19 | cryocare | cryocare – Prediction |
| 20 | cryocare | cryocare – Prepare training Data |
| 21 | cryocare | cryocare – Training |
| 22 | deepfinder | deepfinder - annotate |
| 23 | deepfinder | deepfinder - cluster |
| 24 | deepfinder | deepfinder - display volume |
| 25 | deepfinder | deepfinder - generate sphere target |
| 26 | deepfinder | deepfinder - segment |
| 27 | deepfinder | deepfinder - train |
| 28 | deepfinder | deepfinder - import coordinates |
| 29 | deepfinder | deepfinder - Load Training Model |
| 30 | dynamo | dynamo - bin tomograms |
| 31 | dynamo | dynamo - vectorial picking |
| 32 | dynamo | dynamo - coords to model |
| 33 | dynamo | dynamo - vectorial extraction |
| 34 | dynamo | dynamo - import subtomos from Dynamo |
| 35 | dynamo | dynamo - import tomograms from Dynamo |
| 36 | dynamo | dynamo - model workflow |
| 37 | dynamo | dynamo - subBoxing |
| 38 | dynamo | dynamo - Subtomogram alignment |
| 39 | emantomo | emantomo - align tilt series |
| 40 | emantomo | emantomo - combined refinement |
| 41 | emantomo | emantomo - combined refinement2 |
| 42 | emantomo | emantomo - ctf estimation |
| 43 | emantomo | emantomo - sub-tilt refinement |
| 44 | emantomo | emantomo - average subtomo |
| 45 | emantomo | emantomo - particles extraction from TS |
| 46 | emantomo | emantomo - tomo boxer |
| 47 | emantomo | emantomo - classify subtomos |
| 48 | emantomo | emantomo - tomo boxer convnet |
| 49 | emantomo | emantomo - extraction from tomogram |
| 50 | emantomo | emantomo - tomo fill mw |
| 51 | emantomo | emantomo - tomo initial model |
| 52 | emantomo | emantomo - tomo initial model new |
| 53 | emantomo | emantomo - tomo initial model new2 |
| 54 | emantomo | emantomo - TS align and tomo reconstruction |
| 55 | emantomo | emantomo - subtomogram refinement |
| 56 | emantomo | emantomo - resize tomos |
| 57 | emantomo | emantomo - template matching |
| 58 | gctf | gctf - tilt-series gctf |
| 59 | imod | imod - Apply transformation |
| 60 | imod | imod - Automatic CTF estimation (step 1) |
| 61 | imod | imod - CTF correction |
| 62 | imod | imod - Dose filter |
| 63 | imod | imod - Etomo interactive |
| 64 | imod | imod - Exclude views |
| 65 | imod | imod - Fiducial alignment |
| 66 | imod | imod - Generate fiducial model |
| 67 | imod | imod - Gold bead picker 3D |
| 68 | imod | imod - Import tomo CTFs |
| 69 | imod | imod - Import transformation matrix |
| 70 | imod | imod - Manual CTF estimation (step 2) |
| 71 | imod | imod - Tilt-series normalization |
| 72 | imod | imod - Tomo normalization |
| 73 | imod | imod - Tomo projection |
| 74 | imod | imod - Tomo reconstruction |
| 75 | imod | imod - Xcorr prealignment |
| 76 | imod | imod - X-rays eraser |
| 77 | motioncorr | motioncorr - align tilt-series movies |
| 78 | novactf | novactf - tomo ctf defocus |
| 79 | novactf | novactf - tomo ctf reconstruction |
| 80 | pyseg | pyseg - fils |
| 81 | pyseg | pyseg - graphs |
| 82 | pyseg | pyseg - picking |
| 83 | pyseg | pyseg - 2D classification |
| 84 | pyseg | pyseg - posrec |
| 85 | pyseg | pyseg - preseg membranes |
| 86 | reliontomo | reliontomo - import coordinates 3D from a star file |
| 87 | reliontomo | reliontomo - import subtomograms from a star file |
| 88 | reliontomo | reliontomo - 3D Classification of subtomograms |
| 89 | reliontomo | reliontomo - Tomo CTF refine |
| 90 | reliontomo | reliontomo - De novo 3D initial model |
| 91 | reliontomo | reliontomo - Apply operation to Relion particles |
| 92 | reliontomo | reliontomo - Make pseudo-subtomograms |
| 93 | reliontomo | reliontomo - Sharpen a 3D reference maps |
| 94 | reliontomo | reliontomo - Prepare data for Relion 4 |
| 95 | reliontomo | reliontomo - Reconstruct particle from tilt series |
| 96 | reliontomo | reliontomo - Auto-refinement of subtomograms |
| 97 | reliontomo | reliontomo - Reconstruct particle averaging a set of subtomograms |
| 98 | reliontomo | reliontomo - Tomo frame align |
| 99 | tomo | tomo - 2D particles to subtomograms |
| 100 | tomo | tomo - 2d coordinates to 3d coordinates |
| 101 | tomo | tomo - assign alignment |
| 102 | tomo | tomo - assign tomos to subtomos |
| 103 | tomo | tomo - assign tomograms to tomo masks (segmentations) |
| 104 | tomo | tomo - Tilt-series assign alignment |
| 105 | tomo | tomo - ctf validate |
| 106 | tomo | tomo - consensus classes subtomo |
| 107 | tomo | tomo - import set of coordinates 3D |
| 108 | tomo | tomo - import 3D coordinates from scipion |
| 109 | tomo | tomo - import subtomograms |
| 110 | tomo | tomo - import tomograms |
| 111 | tomo | tomo - import tomomasks (segmentations) |
| 112 | tomo | tomo - import tilt-series |
| 113 | tomo | tomo - import tilt-series movies |
| 114 | tomo | tomo - split even/odd tomos/subtomos |
| 115 | tomo | tomo - extract 3D coordinates |
| 116 | tomo | tomo - tomograms to micrographs |
| 117 | tomo | tomo - average tilt-series movies |
| 118 | tomo3d | tomo3d - denoise tomogram |
| 119 | tomo3d | tomo3d - reconstruct tomogram |
| 120 | tomosegmemtv | tomosegmemtv - annotate segmented membranes |
| 121 | tomosegmemtv | tomosegmemtv - Resize segmented or annotated volume |
| 122 | tomosegmemtv | tomosegmemtv - tomogram segmentation |
| 123 | tomoviz | tomoviz - picking consensus |
| 124 | tomoviz | tomoviz - remove duplicates |
| 125 | tomoviz | tomoviz - filter by normal |
| 126 | xmipptomo | xmipptomo - align transformations |
| 127 | xmipptomo | xmipptomo - apply alignment subtomo |
| 128 | xmipptomo | xmipptomo - apply alignment tilt-series |
| 129 | xmipptomo | xmipptomo - connected components to ROIs |
| 130 | xmipptomo | xmipptomo - cltomo |
| 131 | xmipptomo | xmipptomo - connected components |
| 132 | xmipptomo | xmipptomo - crop tomograms |
| 133 | xmipptomo | xmipptomo - fit vesicles |
| 134 | xmipptomo | xmipptomo - half maps |
| 135 | xmipptomo | xmipptomo - misalign tilt-series |
| 136 | xmipptomo | xmipptomo - local Resolution MonoTomo |
| 137 | xmipptomo | xmipptomo - phantom create subtomo |
| 138 | xmipptomo | xmipptomo - resize tilt-series |
| 139 | xmipptomo | xmipptomo - resize tomograms |
| 140 | xmipptomo | xmipptomo - imagej roi |
| 141 | xmipptomo | xmipptomo - Astigmatism rotation |
| 142 | xmipptomo | xmipptomo - score/filter coordinates |
| 143 | xmipptomo | xmipptomo - score transformations |
| 144 | xmipptomo | xmipptomo - split tilt-series |
| 145 | xmipptomo | xmipptomo - map back subtomos |
| 146 | xmipptomo | xmipptomo - subtomo projection |
| 147 | xmipptomo | xmipptomo - subtomo subtraction |
| 148 | xmipptomo | xmipptomo - tiltseries FlexAlign |
